# Enhancing Duck Manure Anaerobic Digestion with Hydrochar: Exploring Green Material Potential via Bidirectional AD-HTC Coupling

**DOI:** 10.3390/ma19081563

**Published:** 2026-04-14

**Authors:** Li Ren, Xinyan Zhang, Xiaohui Xu, Qingyu Qin, Haotian Fan, Ziliang Wang, Wenlong Wang

**Affiliations:** 1 Shandong Key Laboratory of Green Thermal Power and Carbon Reduction, School of Nuclear Science, Energy and Power Engineering, Shandong University, Jinan 250061, China202414539@mail.sdu.edu.cn (H.F.);; 2 Institute of Animal Science and Veterinary Medicine, Shandong Academy of Agricultural Sciences, Jinan 250061, China; 3 Institute of Environment and Ecology, Tsinghua Shenzhen International Graduate School, Tsinghua University, Shenzhen 518055, China

**Keywords:** hydrochar, anaerobic digestion, duck manure, methane yield, microbial community, metabolic pathway

## Abstract

The efficient resource utilization of duck manure and agricultural/forestry wastes (AFW) plays a significant role in environmental protection and promoting the sustainable development of the economy and society. This study examined the effects of hydrochar derived from AFW in the anaerobic digestion (AD) process, determining the optimal addition ratio. This research systematically investigated the impact of hydrochar on methane yield, as well as changes of short-chain fatty acids, microbial community dynamics, and metabolic pathways during AD of duck manure. The underlying mechanisms were clarified by metagenomic and metabolomic analyses. This experiment used duck manure as substrate and added hydrochar of four different dosage levels. Laboratory batch tests ran for 32 days at 37 ± 0.5 °C, with three parallel samples for each group. The results indicated that hydrochar additive significantly improved methane yield (*p* < 0.05), with a maximum increase of 27.13% at an optimal dosage of 10.91 g·L^−1^. This amendment enhanced the abundance of *Firmicutes*, *Bacteroidota*, *Chloroflexota*, *Halobacteriota,* and *Methanosarcina* significantly. Compared to the control group, the abundances of functional genes involved in hydrolysis, acidogenesis, and acetogenesis pathways increased by 28–254% in the optimal treatment group, with methanogenesis-related genes showing a 16–155% enhancement (*p* < 0.05).

## 1. Introduction

The meat duck output of China reached 4.22 billion heads in 2024, accounting for more than 82% of the worldwide total output [1]. Tai’an City in Shandong Province is the largest meat duck breeding base in China, with an annual meat duck breeding volume reaching 2100 million [2]. Based on the average daily manure production of 0.259 kg per meat duck, approximately 30 million tons of duck manure is produced every year in Tai’an. The rational disposal and consumption of large amounts of duck manure is an urgent challenge for the sustainable development of the meat duck farming industry in Tai’an.

There are significant differences in manure characteristics between meat duck and other livestock. Duck manure is characterized by high water content, high oil content, high ammonia nitrogen, high viscosity, and strong foul odor [3,4,5]. These characteristics determined that AD was the optimum method for duck manure treatment. However, during the AD of duck manure, significant inhibitory effects often occur, accompanied by high nitrogen loss ratios, low energy conversion rates, and difficulties in utilizing the liquid digestate. Ammonia nitrogen concentrations within the range of 200–1500 mg·L^−1^ are favorable for methanogenesis [6], while concentrations around 2500 mg·L^−1^ can inhibit methanogenic bacteria [7]. Ammonia nitrogen concentration can reach 5000–6000 mg·L^−1^ during the AD of duck manure, and ammonia inhibition disrupted the metabolic processes, reduced energy conversion efficiency, led to the accumulation of volatile fatty acids (VFAs), and affected the production of methane [8,9,10]. Therefore, acceleration the AD process to achieve more biogas is essential during the energy conversion recycling of nitrogen-rich substrates [11,12].

Recent studies have indicated that exogenous additives such as biochar, hydrochar, and magnetite can accelerate the process of AD. Among them, hydrochar, the solid production of hydrothermal carbonization (HTC) [13,14,15], could accelerate organic matter hydrolysis [16,17], improve acidogenesis [18], enhance VFA degradation [19,20], and promote methanogenesis during the AD process [21,22]. Therefore, hydrochar has gained significant attention due to its enormous potential in the AD process [23]. The research of Hurst [24] showed that hydrochar from acid hydrolysis reduced ammonium concentration and increased microbial diversity. A recent study [25] found that hydrochar could remove ammonia and promote VFAs converting to biogas during the AD of dead pig carcasses. The research of Ren [26] proved that hydrochar increased hydrogenotrophic methanogenesis and methanogenesis from acetic acid, likely due to direct interspecies electron transfer (DIET).

Although several studies have confirmed the effectiveness of hydrochar on AD, none of them used duck manure as substrate, and the optimal addition ratio of hydrochar showed significant variation across studies. The research of Usman [15] demonstrated that 10 g·L^−1^ hydrochar enhanced methane production in the AD of hydrothermal liquefaction wastewater. The study of Sugiarto [11] revealed that 15 g·L^−1^ hydrochar increased methane yield in the AD of food waste. The investigation of Yu [27] indicated that 120 mg·L^−1^ biochar achieved the highest methane production in the AD of food waste. Therefore, in terms of the effect of hydrochar on the AD of duck manure, the optimal addition ratio required further experimental determination. The mechanisms to promote the inhibition relief and methane production enhancement by porous slow-release and functional group conductive mediators in AD required further investigation.

Based on the above, to dispose of duck manure and other high-nitrogen organic matter, an innovative idea for the bidirectional coupling through HTC and AD is proposed. The liquid digestate of AD can be used as the liquid carrier in the HTC process, and hydrochar can be used as an additive to enhance the AD process, aiming to solve problems such as digestion inhibition, low gas production rate, and inability to dispose of liquid digestate.

In this study, duck manure and hydrochar derived from AFW were used as raw materials to verify the feasibility of the technology based on bidirectional coupling AD and HTC, exploring the effects of hydrochar and the mechanism. This research systematically investigated the impact of hydrochar on methane yield, changes of short-chain fatty acids (SCFAs), microbial community dynamics, and metabolic pathways during the AD of duck manure. The underlying mechanisms were clarified by metagenomic and metabolomic analyses.

## 2. Materials and Methods

### 2.1. Inoculum and Substrate Preparation and Characterization

The inoculum was obtained from a 25 L laboratory anaerobic digester with stable and long-term operation for chicken manure treatment, and then it was quickly concentrated and transferred into this experiment. Fresh duck manure was obtained from Xintai Tianxin Agricultural and Animal Husbandry Development Co., Ltd., Tai’an, China, and the duck manure was collected from the early stage (days 1–7) of a meat duck stereo farming cycle. Before the experiment, duck manure was stored in the refrigerator at −24 °C for no more than 7 days to minimize the degradation of organic matter and changes in physicochemical properties. To further reduce potential bias, all experimental groups used duck manure from the same batch of storage. When starting the experiment, the frozen duck manure was unfrozen to room temperature and stirred evenly. The key characteristics of duck manure and inoculum are summarized in Table 1.

### 2.2. Preparation and Characteristics of Hydrochar

#### 2.2.1. Preparation of Hydrochar

In this study, the AFW was used as feedstock for hydrochar preparation. Before the experiment, the branches were crushed and grounded into particles. These biomass particles were then combined with water at a 1:9 mass ratio and transferred to a 1000 mL HTC reactor; the reactor was constructed of 316 stainless steel and equipped with an automatic temperature control system and stirring controller. The reactor was heated at a rate of approximately 5 °C·min^−1^, maintained at 230 °C for 1 h with a stirring speed of 400 rpm, and subsequently cooled to room temperature by cooling water. The pressure was gradually increased and ultimately reached 2.3 MPa. A vacuum filtering device was then used to separate the liquid and solid product. Solid hydrochar product was finely ground to a particle size under 0.25 mm using a Retsch SM 300 cutting mill (Retsch GmbH, Haan, Germany) with a 0.25 mm sieve insert, then dried at 35 °C for 24 h and stored in a glass-sealed vial.

#### 2.2.2. Characteristics of Hydrochar

**Figure 1:**
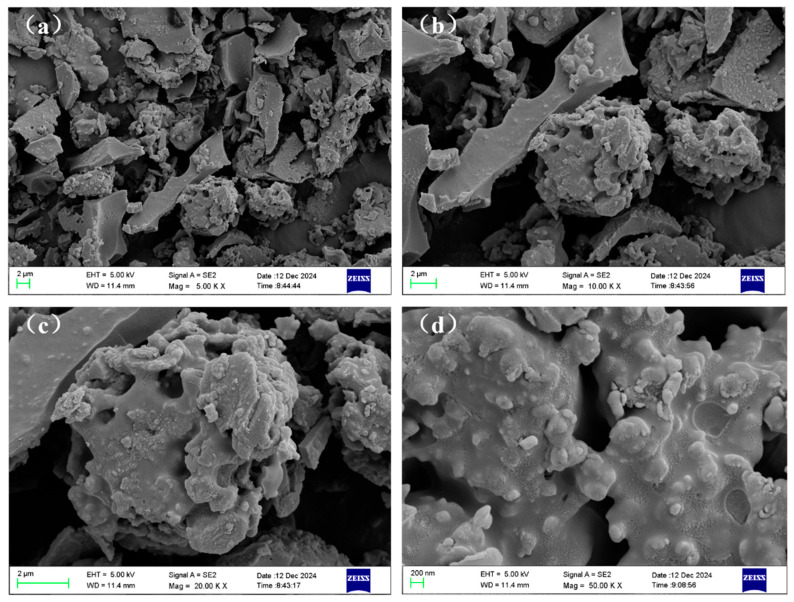
Surface morphology of hydrochar presented using SEM ((**a**) magnification is 5.00 K X; (**b**) magnification is 10.00 K X; (**c**) magnification is 20.00 K X; (**d**) magnification is 50.00 K X).

Table 2 shows the chemical and physical properties of hydrochar. The large specific surface area of hydrochar in this experiment, up to 123.88 m^2^·g^−1^, provided ample space for microbial growth during AD, which was to facilitate the adsorption of ammonium and VFAs. The BET surface area of hydrochar differed based on the type of material utilized. Figure 1 displayed the SEM images illustrating the surface morphology of hydrochar. From lower magnifications (Figure 1a,b), it was shown that the hydrochar had flake-like and quasi-spherical morphologies. Different morphological structures of hydrochar stacked together, forming a rich pore structure. Upon further magnification of the morphology, the surface of the hydrochar was rough and uneven, with numerous pores on its own, thus resulting in a large specific surface area. The surface pores and functional groups of hydrochar can serve as binding sites for the immobilization and enrichment of functional microorganisms, providing more habitats and proliferation space for functional bacteria related to organic matter hydrolysis, acidogenesis, and methanogenesis in the AD system [24,28,29], which is beneficial to methane production.

**Figure 2:**
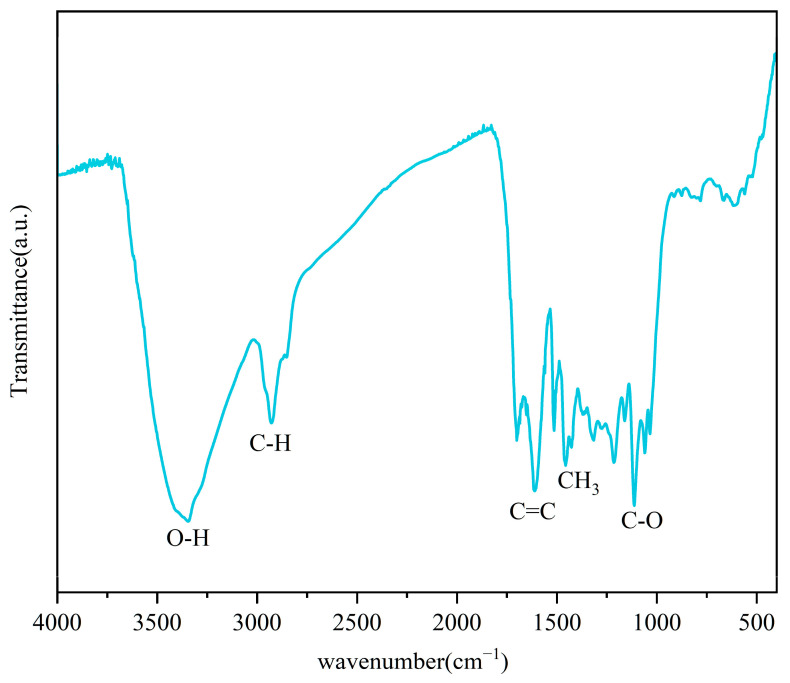
FTIR spectra of hydrochar.

FTIR spectroscopy (Figure 2) indicated the aromatization of hydrochar with peaks at 1513 cm^−1^ and 1602 cm^−1^ corresponding to aromatic C=C bonds. Aromatic deoxygenated surfaces, known for their hydrophobic properties [30], facilitate biofilm formation and enhance microbial activity. The peak at 3340 cm^−1^ corresponded to the O–H of hydroxyl and carboxyl groups, while the peak at 1115 cm^−1^ was associated with C–O bonds. As proved in the research [31], hydrochar had higher peaks relating with C–O and O–H bonds, which was associated with DIET. As shown in the research of Ren [26], hydroxyl groups (-OH) could act as electron shuttles to transfer electrons between electricity-producing bacteria (electron donors) and methanogenic archaea (electron acceptors). Additionally, oxygen-containing functional groups can form charged sites on the hydrochar surface, which can serve as temporary electron storage sites for microbial extracellular electron transfer [26], thereby enhancing the stability of DIET. As demonstrated by previous studies [32], hydrochar enriched microorganisms through surface functional groups, and the enhancement of AD was positively correlated with the abundance of surface oxygen-containing functional groups.

### 2.3. Batch Digestion Test

Batch experiments were conducted in high-borosilicate glass bottles with a nominal volume of 500 mL, which were separated into control groups and hydrochar treatment groups. To adjust for methane yield, the group C0 was fed with 220 mL inoculum only as a blank group, and the group C1 was fed with 22 g duck manure and 220 mL inoculum without hydrochar as a control group. The headspace volume of each bioreactor was 363 mL. In the hydrochar groups, each bioreactor was fed with 22 g duck manure and 220 mL inoculum. The F/M value based on the VS of each group was 17.0. The hydrochars of 0.06 g, 0.12 g, 0.24 g, and 0.36 g were added to each bioreactor separately, and the hydrochar to duck manure ratios of HC1, HC2, HC3, and HC4 groups were 2.73 g·L^−1^, 5.45 g·L^−1^, 10.91 g·L^−1^, and 16.36 g·L^−1^, respectively.

All bioreactors were sealed with butyl rubber stoppers, purged with nitrogen for four minutes, and the oxygen concentration in the bioreactor was below 0.5% to ensure the anaerobic environment. Then, the bioreactors were put in an air bath with a shaker operating at 120 rpm to guarantee a steady digestion temperature of 37 ± 0.5 °C. The batch AD experiments lasted about 32 days. Three parallel samples were set up for each group to ensure the reliability of the data.

Biogas was collected by the water displacement method, and the biogas volume was determined by measuring the volume of discharged water. Biogas samples were taken every 4 days to measure cumulative biogas production and methane concentration. The produced biogas was stored in air bags. Additionally, biogas components and concentrations of SCFAs were determined every 7 days.

The calculation formula for methane yield per group is as follows.

(1) $$M_{{CH}_{4}}=\frac{M_{\mathrm{biogas}}\times C_{{CH}_{4}}-M_{\mathrm{biogas},\mathrm{blank}}\times C_{{CH}_{4},blank}}{M_{substrate}\times \mathrm{TS}\times \mathrm{VS}}$$

where M*_CH_*_4_ is daily methane yield (mL·g^−1^ VS) per group, M*_biogas_* is daily biogas yield (mL) of each group, M*_biogas,blank_* is daily biogas yield (mL) of the blank group, C*_CH_*_4_ is the volume concentration of methane in biogas, and M*_substrate_* is the weight of substrate of each group.

### 2.4. Analytical Methods

The main elemental compositions of hydrochar were determined by an elemental analyzer (SDCHN636, Hunan Sande Technology Co., Ltd., Changsha, China). The Mad, Aad, Vad, and FCad of hydrochar were analyzed using an industrial analyzer (SDTGA6000, Hunan Sande Technology Co., Ltd., Changsha, China). The average pore size and specific surface area of hydrochar were measured by the specific surface area and pore size analyzer (JW-BK100, JWGB Sci. & TECH. Co., Ltd., Beijing, China). The micro morphology of hydrochar was scanned using a thermally assisted field scanning electron microscope (SEM) (Supra 55, Zeiss RMS, Oberkochen, Germany). The surface functional groups of hydrochar were determined by a Fourier transform infrared spectrometer (FTIR spectrometer) (Nicolet 6700, Thermo Fisher, Waltham, MA, USA).

The biogas yield was measured using the wet flowmeter (LMF-1) (LMF-1, Beijing Jinzhiye Instrument Equipment Co., Ltd., Beijing, China). Gas chromatography (A91 Plus, Changzhou Pannor Instruments Co., Ltd., Changzhou, China) was used to measure the composition of biogas. The concentration of SCFAs was estimated as the sum of ethanol, acetic acid, propionic acid, n-butyric acid, isobutyric acid, n-valeric acid, and isovaleric acid, and it was evaluated by gas chromatography, which was equipped with a thermal conductivity detector and argon as the carrier gas (30 mL·min^−1^). The temperatures of the column oven, injector, and detector were 50 °C, 150 °C, and 180 °C, respectively. The VS and TS were measured using the APHA standard methods [33].

### 2.5. Metagenomic Analysis

Digestate samples obtained from the C1, HC1, HC2, HC3, and HC4 at the 12th day of AD were used for metagenomic sequencing and analysis. The 12th day was selected as the sampling time point based on the methane production kinetics, which showed the highest methane yield at this stage. This timing allowed us to analyze the microbial community and functional gene abundance corresponding to the peak methanogenic activity. To ensure the reliability of analysis data, three parallel tests were conducted for each sample.

To extract microbial genomic DNA samples, the OMEGA Mag-Bind Soil DNA Kit (M5635-02) (Omega Bio-Tek, Norcross, GA, USA) was utilized. The amount of DNA obtained was evaluated by a Qubit™ 4 Fluorometer, with WiFi: Q33238 (Qubit™ 1X dsDNA HS Assay Kit: Q33231; Qubit™ Assay Tubes: Q32856) (Invitrogen, Carlsbad, CA, USA), and agarose gel electrophoresis was performed to determine the quality. The microbial DNA was inserted into fragments with sizes around 400 bp. At Personal Biotechnology Co., Ltd. (Shanghai, China), samples were extracted and shotgun sequenced by the Illumina NovaSeq platform (Illumina, San Diego, CA, USA).

The raw sequencing reads were processed to obtain quality-filtered reads for further analysis. First, sequencing adapters were eliminated from the reads using Cutadapt (v1.2.1) [34]. Second, low-quality reads were trimmed via a sliding-window algorithm in fastp (v0.23.2). Kraken2 (v2.0.8-beta) was used to taxonomically classify the metagenomics sequencing reads [35] against an GTDB-derived database. Megahit (v1.1.2) was employed to assemble reads from each sample with the meta-large preset parameters [36]. Contigs greater than 300 bp were merged and clustered with mmseqs2 [37] in the “easy-linclust” mode, with a sequence identity threshold of 0.95 and 90% coverage of the shorter contig’s residues. To determine the lowest common ancestor taxonomy of non-redundant contigs, they were aligned against the NCBI-nt database using mmseqs2 in ‘taxonomy’ mode; contigs classified as Viridiplantae or Metazoa were excluded from subsequent analyses. Gene prediction was generated by Prodigal (V2.6.3) [38].

The CDS sequences of all samples were clustered using the mmseqs2 software in “easy-cluster” mode, with a protein sequence identity threshold of 0.95 and a required coverage ratio of 90% for shorter contigs. To evaluate the abundance of these genes, high-quality sequencing reads from each sample were mapped to the predicted gene sequences using Minimap2 with the parameters “-ax sr --sam-hit-only”, and featureCounts was used to count the number of reads aligned to the gene sequences, namely, the read count (RC) for each gene [39]. The functions of non-redundant genes were annotated by searching the KEGG protein database using mmseqs2 software in “search” mode.

### 2.6. Statistical Analysis

Each measurement was performed in triplicate at a minimum, and data are presented as the mean ± standard deviation. Significant differences analysis was performed using SPSS 25.0 (IBM Corporation, Chicago, IL, USA) with analysis of variance (ANOVA). For the abundances of microbial community and functional genes, the Kruskal–Wallis (KW) test was conducted followed by log10 data transformation and Benjamini–Hochberg (BH) multiple testing correction to reduce the false positive impact caused by the direct use of relative abundance. When the *p*-value was <0.05 (*), *p* < 0.01 (**), or *p* < 0.001 (***), there was a significant difference between the two groups. All graphs were constructed using OriginPro 2024 (OriginLab Corporation, Northampton, MA, USA).

## 3. Results and Discussion

### 3.1. Impact of Hydrochar on the AD of Duck Manure

#### 3.1.1. Influence of Hydrochar on Methane Yield

**Figure 3:**
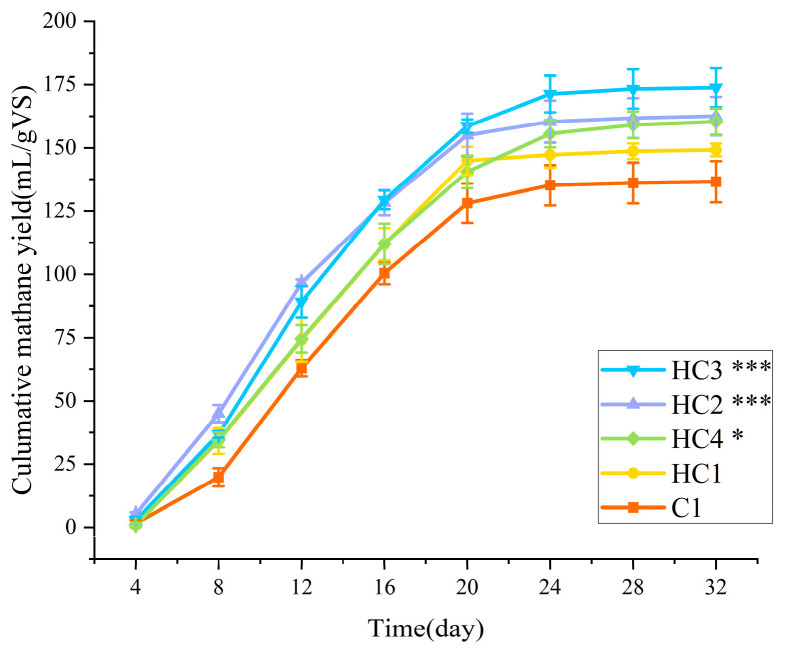
Cumulative methane yield of all the groups. Significance is denoted as *p* < 0.05 (*), *p* < 0.001 (***).

The cumulative methane yields of the five groups (not including C0 only) during 32-day AD are shown in Figure 3. After deducting the influence of inoculum (C0), the cumulative methane yields of C1, HC1, HC2, HC3, and HC4 reached 138.17 ± 3.99, 149.95 ± 2.62, 161.52 ± 3.89, 175.65 ± 1.84, and 158.17 ± 3.83 mL·g^−1^ VS, respectively. The highest methane yield (175.65 mL·g^−1^ VS) was obtained at HC3, which led to a 27.13% increase compared to C1. With the adding of hydrochar, the methane yield was significantly higher than that of the control group (*p* < 0.05), which proved the accelerating ability of hydrochar on methane production. The optimal dosage of hydrochar was largely consistent with those reported by Usman [15] and Sugiarto [11]. Shen [40] mentioned that the increase in methane content could be due to the transformation of VFA and ethanol to methane by the functioned groups that exist on the surface of hydrochar and that the alkaline nature of the hydrochar maintained the optimal pH for anaerobic digestion. The methane yield initially increased with the growth of hydrochar dosage but subsequently declined with excessive addition, which aligns well with the observations of Choe [18] and Xu [25]. It is hypothesized that the excessive hydrochar adsorbed additional VFAs prior to biogas generation, thereby reducing the available substrate for methanogenesis. Additionally, more hydrochar particles adhere to the surface of the substrate, decreasing the contact area between methanogenic bacteria and the substrate [25].

**Figure 4:**
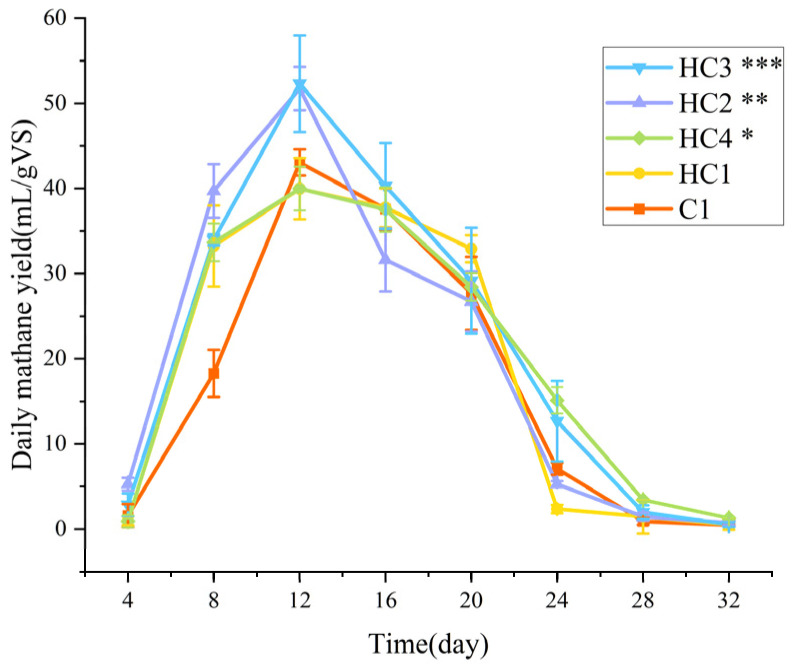
Daily methane yield of all the groups. Significance is denoted as *p* < 0.05 (*), *p* < 0.01 (**), *p* < 0.001 (***).

Figure 4 exhibits the daily methane yield of all the five groups during 32-day AD (*p* < 0.05). The variation trends of daily methane yield in all groups were similar, which first increased (4–12 days) and then decreased (12–28 days), and the daily methane yield of all the groups reached close to 0 in the final stage (28–32 days). The methane yield of all groups reached their respective peaks on the 12th day. Compared with the other groups, the dosages of hydrochar in HC2 and HC3 were beneficial for the optimal sustainability of methane production. When hydrochar was added into AD system, its substantial specific surface area and well-developed porous established a three-dimensional scaffold structure. This structure provided expanded colonization habitats for methanogenic archaea, resulting in rapid increasing of methane production rates.

#### 3.1.2. Effect of Hydrochar on Concentration and Composition of SCFAs During AD

**Figure 5:**
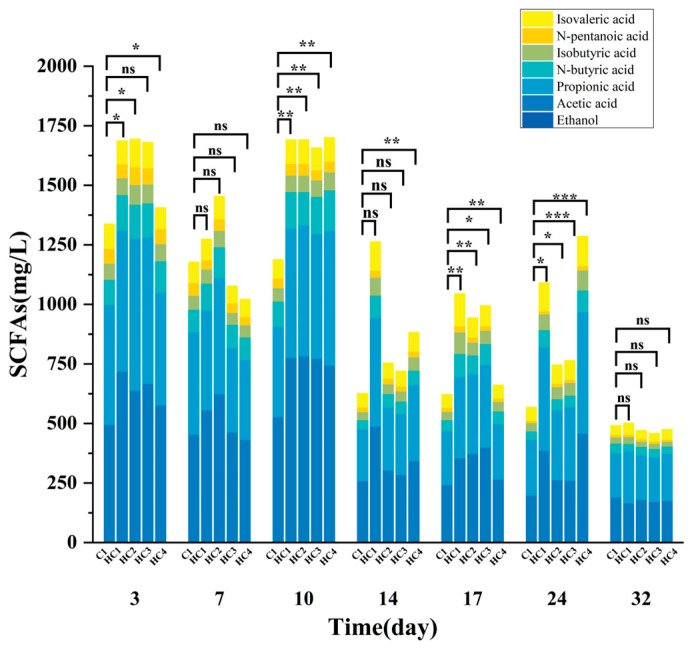
SCFA concentration and composition of all the groups. Significance is denoted as *p* < 0.05 (*), *p* < 0.01 (**), *p* < 0.001 (***), “ns” indicates not significant.

As shown in Figure 5, the concentration and composition of SCFAs were significantly affected by hydrochar during the AD of duck manure. Compared to C1, hydrochar increased the concentration of SCFAs during the 3 to 24 days, which could be attributed to the promotion of hydrolysis and acidiogenesis processes. Between days 25 to 32, the consumption of organic matter was nearly complete, and hydrochar decreased the concentrations of total SCFAs at last.

On the third day, hydrochar groups had higher concentrations of SCFAs, indicating that hydrochar may accelerate hydrolysis and acidogenesis of AD. The concentrations of SCFAs in hydrochar groups reduced dramatically on the seventh day compared to the third day, indicating that hydrochar may stimulate SCFA breakdown, hence speeding up the methanogenesis process. It was noteworthy that the degradation rates of SCFAs in HC1 and HC4 were lower than those in HC2 and HC3, which was consistent with the methane yield. A resent study [41] showed similar trends in SCFA changes, further supporting that hydrochar has the ability to promote the decomposition of SCFAs.

As various acids converted into methane under the action of hydrogenotrophic methanogens and acetoclastic methanogens during the methanogenesis process, hydrochar addition increased the concentration of SCFAs and promoted the composition of SCFAs, thereby leading to more and faster methane production.

Excess hydrochar (HC4) addition resulted in lower SCFA levels during early fermentation, probably because excess hydrochar adsorbed more SCFAs prior to biogas formation, reducing available substrates and causing a minor decline in methane production. This finding aligns with the conclusions [25].

#### 3.1.3. Effect of Hydrochar on Organic Matter Degradation

**Figure 6:**
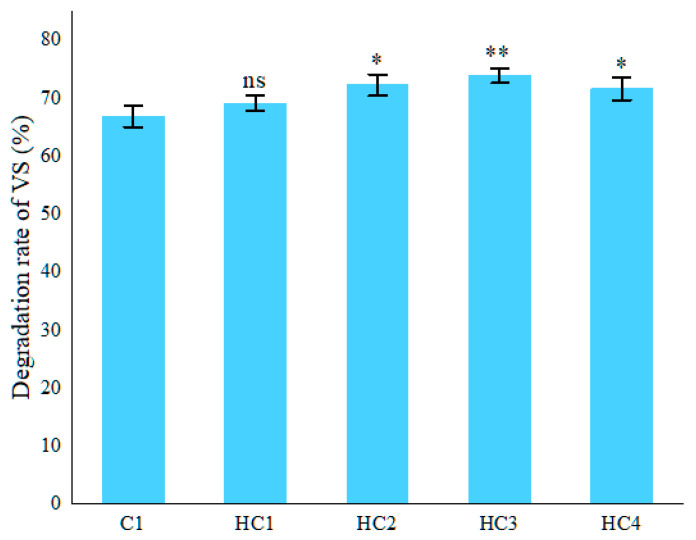
Degradation rates of VS of all the groups. Significance is denoted as *p* < 0.05 (*), *p* < 0.01 (**), “ns” indicates not significant.

The initial F/M ratio indicated that the substrate supply was relatively sufficient, providing abundant metabolic substrates for methanogens. By using the same F/M ratio for all the groups, differences in substrate load were excluded as a factor interfering with the experimental results. Figure 6 shows the VS degradation rates of all the groups with or without hydrochar. The VS degradation rate of C1 was 66.62%, and the VS degradation rates of HC1, HC2, HC3, and HC4 were 69.21%, 72.32%, 73.95%, and 71.69%, which were 3.88~11.00% higher than C1. The VS degradation rate increased with the addition of hydrochar, reaching its maximum value in HC3. However, when an excessive amount of hydrochar was added, the VS degradation rate decreased in HC4.

Therefore, hydrochar could effectively enhance the degradation of VS, and then increase the methane yield. The order of VS degradation rates from high to low was consistent with that of methane yield, indicating a strong positive correlation between VS degradation efficiency and methane yield.

### 3.2. Changes of Microbial Community

#### 3.2.1. Diversity of Microbial Community

The total number of original reads for all samples ranged from 8.97 × 10^7^ to 1.35 × 10^8^. After quality filtering, a total of 8.91 × 10^7^ to 1.34 × 10^8^ clean reads were obtained per sample, with an average of 1.34 × 10^8^ clean reads per sample. The average sequencing depth was sufficient for metagenomic binning analysis, and the effective sequencing coverage of all samples ranged from 98.53% to 99.31%, which indicated that the sequencing data quality met the requirements for subsequent microbial genome reconstruction, community composition, and functional annotation analysis.

**Figure 7:**
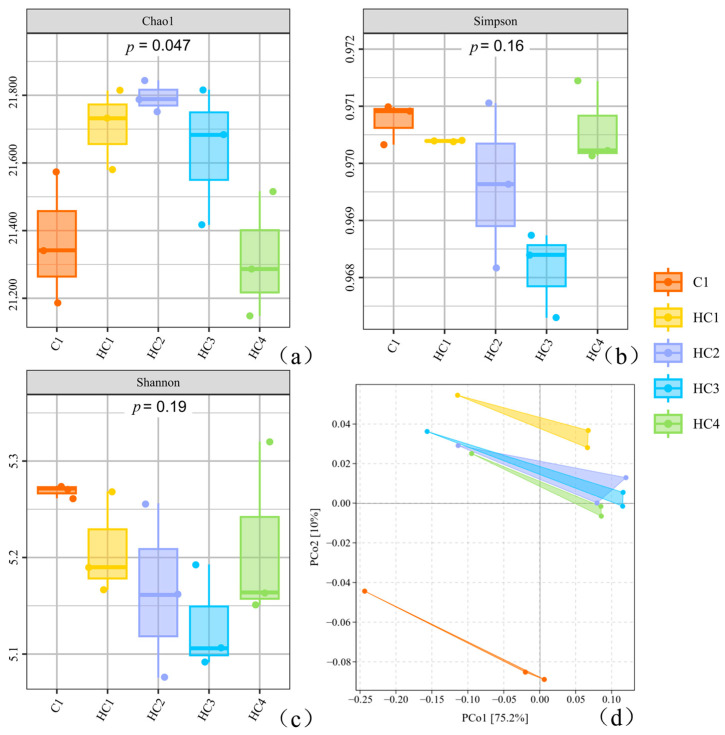
The diversity of microbial community based on bacterial coverage ((**a**) Chao index, (**b**) Simpson index, (**c**) Shannon index, (**d**) PCoA analysis).

The addition of hydrochar significantly modified the microbial community structure (Figure 7). The Chao index (Figure 7a) indicated higher community richness in the hydrochar groups compared to C1. The Simpson index (Figure 7b) and Shannon index (Figure 7c) indicated a significant decrease and wider range in community diversity and richness with hydrochar addition, potentially due to changes in community structure. As shown in Figure 7d, adonis analysis based on Bray–Curtis distance was performed to test the differences between groups, and the results showed no significant differences among groups. The two coordinates contributed to more than 80% of total variance (PCo1 75.2% and PCo2 10%), and the research revealed that the hydrochar and control groups were significantly separated, indicating that hydrochar addition imposed selection pressure on the microbial population.

#### 3.2.2. Microbial Community Structure

**Figure 8:**
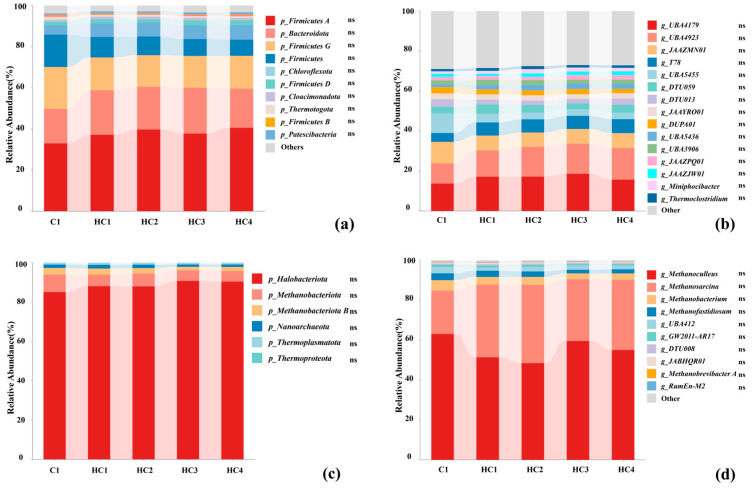
Analysis of microbial community ((**a**) the relative abundance of the top 10 bacteria at the phylum level, (**b**) the relative abundance of the top 15 bacteria at the genus level, (**c**) the relative abundance of archaea at the phylum level, (**d**) the relative abundance of the top 10 archaea at the genus level). Significance was denoted as ns: not significant.

All MAGs underwent genome integrity and contamination assessment using CheckM v1.1.3. MAGs with a screening integrity ≥ 50% and contamination < 10% were selected for subsequent analysis. All MAGs reported in the results met the screening criteria. A total of 144 MAGs were annotated to species, 140 MAGs were recognized as bacteria and categorized into 17 phyla, and 4 MAGs were identified as archaea and categorized into 2 phyla, according to the GTDB-tk annotation results. The microbial communities in the digestate from all the groups were characterized in Figure 8, and detailed data are provided in Table S1. Kruskal–Wallis (KW) test results showed that there were no significant differences in the abundance among the five groups.

As shown in Figure 8a, the bacteria composition in all groups was predominantly comprised of five phyla: *Firmicutes*, *Bacteroidota*, *Chloroflexota*, *Cloacimonadota*, and *Proteobacteria*. *Firmicutes*, *Chloroflexota*, *Bacteroidetes*, and *Proteobacteria* played important roles in the hydrolysis and acidogenesis process [42,43]. The relative abundance of *Firmicutes A* ranged from 37.15% to 40.57% in the hydrochar groups, while the lowest abundance in C1 was 32.97%. The relative abundance of *Bacteroidota* peaked in HC3 at 30.81% and was lowest in C1 at 23.01%. The relative abundance of *Chloroflexota* ranged from 7.49% to 8.25% in the hydrochar groups, while the lowest abundance in C1 was 5.18% (*p* < 0.05). *Firmicutes* was the primary degrader in the early fermentation stage, responsible for degrading various sugars and hydrocarbons to pre-methane compounds such as acetic acid, H_2_, and CO_2_, serving as a critical link between hydrolysis and methanogenesis processes [44]. *Bacteroidota*, which was reported to have the ability to degrade nitrogenous and phenolic organics [45], could break down sugars and amino acids, producing succinate and acetate [46,47]. Additionally, previous studies [48,49] have also documented the function of *Chloroflexi* as a possible electroactive bacterial partner in DIET to promote methanogenesis, and the increased abundance of *Chloroflexota* in hydrochar groups suggested a potential association with DIET-related processes in this study.

A deeper analysis was conducted at the genus level to gain insight into the changes in microbial community induced by hydrochar. The bacterium *g_UBA4179* of family *Dysgonomonadaceae*, phylum *Bacteroidota*, the bacterium *g_UBA 4923* of *Order Saccharofermentanale*, phylum *Firmicutes*, and the bacterium *g_T78* of family *Anaerolineaceae*, phylum *Chloroflexota*, were the three most abundant genera in hydrochar groups, particularly in HC3 (Figure 8b). They all performed crucial roles in hydrolysis and acidogenesis processes, and their high abundance in hydrochar groups could explain the rapid degradation of VS, which was related to the rapid generation of methane at the pre-stage of AD.

As shown in Figure 8c, archaea belonging to the phylum *Halobacteriota* were the dominant archaea in all the groups, with their relative abundance peaking at 90.75% in HC3 and reaching its lowest at 85.04% in C1. According to the research of [50], *methanogens* within the *Halobacteriota* phylum, which contain b-type cytochromes, exhibited higher growth yield and utilized a wider range of substrates compared to those lacking b-type cytochromes. The abundance of *Halobacteriota* might account for the increased methane production observed in hydrochar groups, indicating a potential correlation between the abundance of this phylum and methane production capacity.

As shown in Figure 8d, the hydrochar groups had more methanogens than C1, with higher abundance of *Methanosarcina* and lower abundance of *Methanoculleus* compared to C1. The relative abundances of *Methanosarcina* in hydrochar groups were raised to 30.99–39.18%, compared to 27.13% in C1 (*p* < 0.05). As acetoclastic methanogens, *Methanosarcina* could directly utilize acetate for methane production, whereas *Methanoculleus* was hydrogenotrophic, relying on H_2_/CO_2_ substrates. The decreased abundance of *Methanoculleus* might reduce substrate competition for hydrogenotrophic pathways, indirectly promoting the dominance of acetoclastic pathways.

*Methanosarcina*, a genus of methanogens capable of growing on a wide range of substrates including H_2_, CO, acetic acid, formate, methanol, and methylamine [51,52], predominates throughout the AD process. The increased abundance of *Methanosarcina* in hydrochar groups implied a potential involvement of this genus in DIET-related electron transfer [53]; however, further exploration of metagenomic evidence is needed to confirm DIET activity. Its high abundance might accelerate the conversion of acetate to methane, effectively preventing acetate accumulation and acidification inhibition, which was positively correlated with the reduced acetate accumulation and higher methane yield, suggesting a potential role in mitigating acidification inhibition. Additionally, *Methanosarcina* can adapt to high ammonia nitrogen and high organic loading environments through metabolic regulation [52,54]. The higher abundance of *Methanosarcina* in hydrochar groups suggested a potential adaptation to the high ammonia nitrogen environment of duck manure AD; however, further investigation into the actual metabolic activity of acetate lyase is needed.

### 3.3. Metabolism Pathways and Related Functional Genes

#### 3.3.1. Analysis of Associated Genes in Hydrolysis, Acidogenesis, and Acetogenesis Pathways

**Figure 9:**
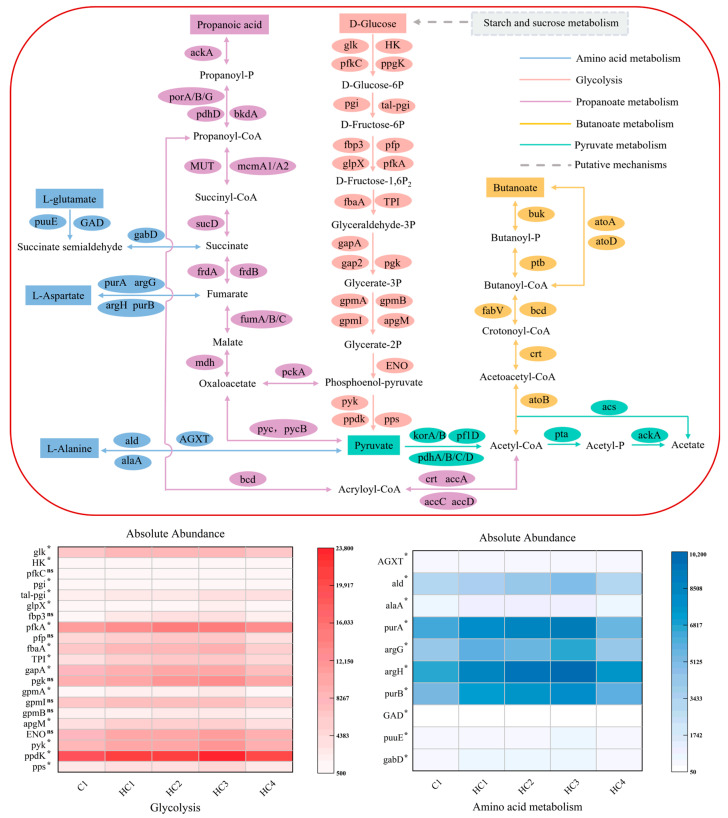

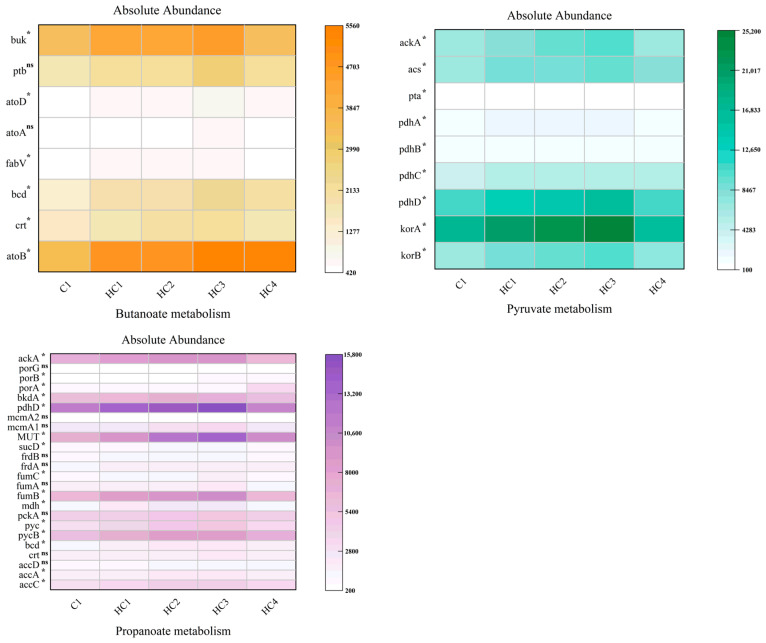
Hydrolysis, acidogenesis, and acetogenesis pathways and associated genes. Significance is denoted as *p* < 0.05 (*); ns: not significant.

During the hydrolysis process, proteins and lipids of duck manure were decomposed into amino acids and glucose, which were further transformed into various acids through the acidogenesis process. Subsequently, various acids, containing pyruvate, butanoate, and propanoate, were broken down into acetic acid by the acetogenesis process (Figure 9). The detailed data are provided in Table S2. Significant differences are shown in Figure 9 based on the Kruskal–Wallis (KW) test.

Firstly, hydrochar enhanced the abundances of all functional genes involved in the glycolysis process, except for gpmB and pfkC. Compared to C1, the absolute abundances of most genes related to glycolysis in HC3 were significantly increased, ranging from 12% to 102%. Gene pps and ppdK participated in substrate activation in the glycolytic pathway; pyk and ENO were core catalytic enzymes of glycolysis; gpmA and pgk were responsible for energy and substance transport during glycolysis. Increased gene abundance suggested enhanced carbohydrate hydrolysis capability and improved production of intermediate metabolic products.

Additionally, hydrochar increased the absolute abundance of functional genes in the amino acid metabolism. The abundance of the associated genes in HC3 was increased from 11% to 55% compared with C1 and was the highest among all hydrochar groups (Figure 9). The metabolic process of amino acids not only produced core methanogenic substrates such as acetic acid and propionic acid but also regulated the system pH and maintained metabolic balance [55]. The synergistic significant upregulation of the aforementioned genes in the HC3 group enhanced the potential supply of diverse methanogenic substrates through amino acid degradation.

The acidification stage, mainly containing pyruvate, propanoate, and butanoate metabolisms, served as a critical link connecting hydrolysis and methanogenesis, providing essential substrates and energy support for methanogenic metabolism. Butyrate, a key AD intermediate, can be further converted to acetic acid for methanogenic utilization. The atoA/B/D genes encode acetyl-CoA transferase (involved in short-chain fatty acid transport/metabolism), crt and bcd regulate fatty acid β-oxidation, and ptb and buk encode phosphotransbutyrate kinase and butyrate kinase (key enzymes in butyrate synthesis). Synergistic upregulation of these genes in HC3 not only enhanced the potential efficiency of fatty acid-to-butrate conversion but also promoted the potential of butyrate into acetic acid through the β-oxidation pathway. This dual mechanism might increase the supply of core substrates for methanogenesis while avoiding toxic inhibition caused by excessive VFAs accumulation.

The acidification stage, dominated by pyruvate, propanoate, and butanoate metabolisms, is a critical link between hydrolysis and methanogenesis, providing essential substrates and energy for methanogenesis. In butanoate metabolism, atoA/B/D genes encode acetyl-CoA transferase (involved in short-chain fatty acid transport/metabolism), crt and bcd regulate fatty acid β-oxidation, and ptb and buk encode phosphotransbutyrate kinase and butyrate kinase (key enzymes in butyrate synthesis). Butyrate, a key anaerobic digestion intermediate, can be further converted to acetic acid for methanogenic utilization. The increased abundance of these key genes in hydrochar groups suggested an elevated potential for butanoate metabolism, which showed a positive correlation with the efficient SCFA consumption and methane production.

Most key pyruvate metabolism genes (e.g., korA, pdhD, acs, ackA) had higher abundances in all hydrochar groups than in C1, showing an initial increase followed by a decrease with increasing hydrochar dosage (*p* < 0.05), with HC3 exhibiting peak abundances in most pathways. Specifically, core pyruvate dehydrogenase complex enzymes (pdhA, pdhB, pdhC, pdhD), kor-series enzymes (korA, korB, mediating pyruvate-to-acetyl-CoA conversion), and acetate-forming genes (acs, ackA) were maximally upregulated in HC3. These results indicated that hydrochar upregulated key pyruvate metabolism gene abundance, enhancing substrate conversion potential [27].

In propanoate metabolism, hydrochar generally upregulated most key genes’ abundances compared to C1, with HC3 showing peak or near-peak abundances across critical pathways. Specifically, pycB, ackA, fumB, MUT, pckA, mcmA, and mdh reached their maximum abundances in HC3. This suggested that hydrochar increased the metabolic potential of the propanoate pathway by elevating the abundance of key genes, which might contribute to the generation of methanogenic precursors and electron carriers.

In conclusion, the upregulation of functional gene abundances in hydrochar groups indicated an elevated metabolic potential in hydrolysis, acidogenesis, and acetogenesis pathways, and these gene abundance changes were positively correlated with the improved AD performance (higher VS degradation, efficient SCFA consumption, increased methane yield).

#### 3.3.2. Analysis of Associated Genes in Methanogenesis Pathways

**Figure 10:**
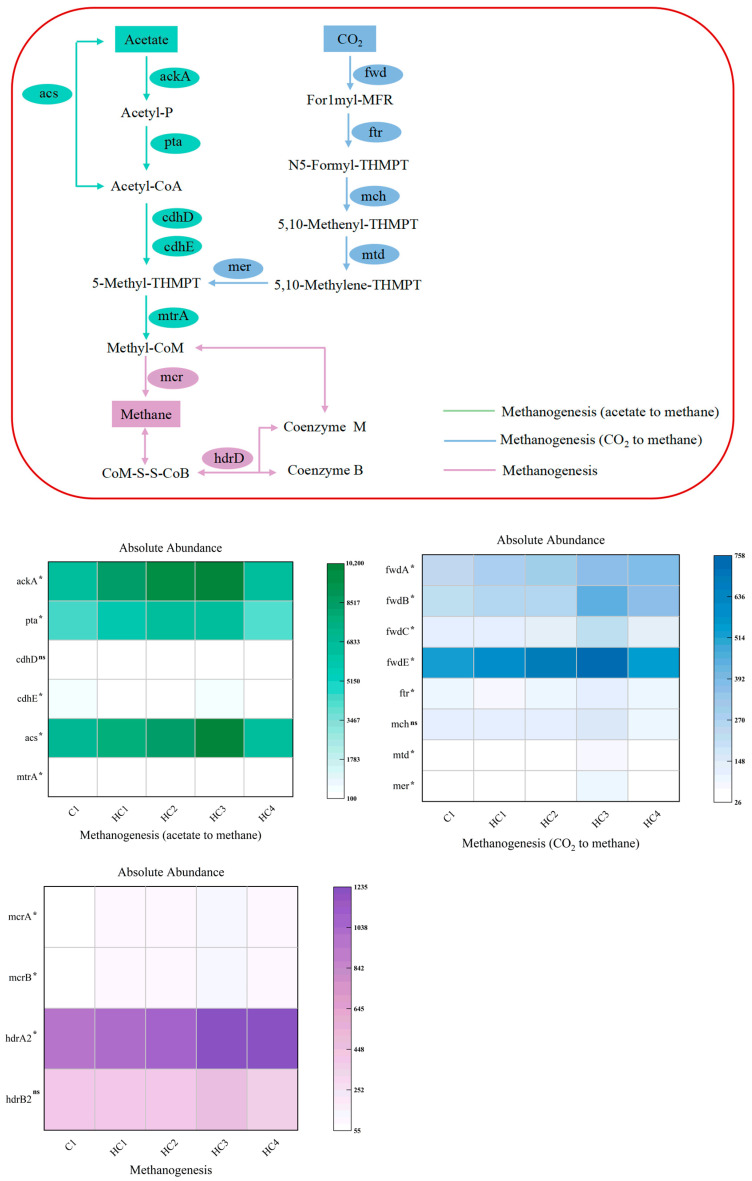
Methanogenesis pathways and associated genes. Significance is denoted as *p* < 0.05 (*); ns: not significant.

The methanogenesis pathways mainly include two pathways: hydrogenotrophic methanogenesis and acetoclastic methanogenesis. The primary methanogenesis pathways and key genes based on the KEGG database are shown in Figure 10, and the data are provided in Table S3. Significant differences are shown in Figure 10 based on the Kruskal–Wallis (KW) test. In particular, the abundances of acs, cdhD, pta, and ackA in HC3 were elevated by 45.0%, 30.1%, 44.1%, and 51.6%, respectively.

Compared to C1, hydrochar generally upregulated the abundances of core hydrogenotrophic methanogenesis genes, including mer, mtd and mch, as well as the subunits of formylmethanofuran dehydrogenase complex (fwdA, fwdB, fwdC, fwdE). All the genes’ abundances peaked in HC3; thereinto, mer, mtd, and fwdB in HC3 increased by the range of 199.0% to 112.8% compared to C1, while the abundances of the other genes were increased from 9% to 87.3%. This finding indicated a significantly elevated abundance of key genes involved in the initial step of hydrogenotrophic methanogenesis, suggesting a higher metabolic potential for the conversion of CO_2_ to formylmethanofuran.

Additionally, mer, which mediates the downstream steps of the methanogenesis pathway, also reached its highest expression level in the HC3 group, further supporting efficient methane production. Collectively, these results demonstrated that the hydrochar dosage applied in HC3 effectively upregulated the abundance of key genes involved in the hydrogenotrophic methanogenesis pathway. The elevated key gene abundance might potentially contribute to improving substrate utilization efficiency and electron transfer efficiency, which could ultimately explain the highest methane production rate observed in HC3.

Mcr and hdr are key genes in the methanogenesis process, playing crucial roles in converting methyl-coenzyme M to methane. Compared to C1, the hydrochar groups showed significantly higher abundances of mcrA and mcrB (*p* < 0.01), with hdrA1 and hdrA2 also significantly increased in HC3 (*p* < 0.01). In the HC3 group, the abundances of mcrA and mcrB increased by 2.1–2.2 times, while the abundances of hdrA2 and hdrB2 increased by 1.2–1.3 times. As the results showed, the terminal methanogenesis step (mcrA/mcrB) was more sensitive to hydrochar than the electron transfer system (hdrA2/hdrB2). The elevation in the abundance of these key methanogenesis genes was positively correlated with the highest methane production in HC3.

The results demonstrated that hydrochar increased the abundance of functional genes involved in both acetoclastic and hydrogenotrophic methanogenesis pathways, indicating an elevated metabolic potential for methane production through these two pathways, and this gene abundance change was positively correlated with the enhanced methane yield in hydrochar groups.

### 3.4. Limitations and Future Research Directions

It should be noted that this study only conducted metagenomic analysis, and the research results exhibit certain representativeness with respect to the microbial community composition and functional gene distribution associated with hydrochar on AD. However, metatranscriptomic was not performed, so the potential DIET mechanism inferred from the increased abundance of microbes and functional genes cannot be confirmed as a causal factor for enhanced methane production. Future research combining metatranscriptomics is needed to confirm the actual metabolic activity of microorganisms, the expression of functional genes at the transcript/protein level, and the causal relationship between DIET and enhanced methanogenesis in hydrochar-amended duck manure AD systems.

This study primarily explored the changes in microbial structure and functional gene abundance induced by hydrochar, but it did not conduct systematic measurement and analysis of carbon flow during AD. As a result, this study was unable to quantify the specific impact of hydrochar on methane carbon, carbon dioxide carbon, liquid carbon, and solid carbon. Therefore, the mechanism by which hydrochar promotes AD through regulating carbon flow still needs to be further explored.

Another limitation of this study is the single-time-point sampling strategy. While day 12 represented the peak methane production stage, future studies with multiple sampling time points would provide more comprehensive insights into the dynamic changes in microbial community structure and function throughout the entire anaerobic digestion process.

As a batch-scale investigation, this study has inherent limitations. Specifically, further experiments focusing on the performance of hydrochar in continuous pilot-scale AD systems are currently in progress to enhance the practical applicability and generalizability of the research findings. Moreover, the effects of different hydrochar types (e.g., derived from different raw materials or prepared under varied HTC conditions) on duck manure AD, under different organic loading rates, remain to be systematically explored in future research.

## 4. Conclusions

This study investigated the mechanism of hydrochar derived from AFW in AD of duck manure. Hydrochar addition in AD of duck manure dramatically increased methane yield by maximum of 27.13%, promoted the consumption of SCFAs, leading to faster methane production. The greatest methane yield was achieved when the dosage of hydrochar to duck manure was 10.91 g·L^−1^. The methane yield initially increased with the growth of hydrochar dosage but subsequently declined with excessive addition.

Metagenomic analysis revealed that the increased abundances of microorganisms and key enzymes in different groups were generally consistent with the corresponding methane production. Hydrochar significantly increased the relative abundances of phyla *Firmicutes*, *Bacteroidota*, and *Chloroflexota*, which played crucial roles in hydrolysis and acidification processes, and the elevated abundance of these phyla in hydrochar groups indicated a higher potential for the hydrolysis and acidification stages of duck manure. The addition of hydrochar increased the abundances of *Halobacteriota* and *Methanosarcina*, which were core taxa for methanogenesis. Furthermore, the abundance of key functional genes involved in hydrolysis, acidogenesis, acetogenesis, and methanogenesis pathways was significantly increased in the hydrochar groups, with the highest levels observed in HC3; this indicated an elevated metabolic potential for these key AD pathways in hydrochar groups.

This experiment verified that the innovative idea of bidirectional coupling HTC and AD is feasible, determined the optimal dosage of hydrochar, and explored the promoting mechanism using metagenomic analysis. Hydrochar might enhance methanogenesis by reshaping microbial community structure and elevating the metabolic potential of key AD pathways through increased functional gene abundance, with the potential DIET effect proposed as a hypothetical underlying mechanism that requires further validation. The technology provides a promising approach to address the bottleneck of low biogas production rate in AD of duck manure, while also offering a potential pathway for biogas liquid utilization.

## Figures and Tables

**Table 1:** Characteristics of duck manure and inoculum.

Parameters	Duck Manure	Inoculum
TS (%)	22.73 ± 2.17	4.31 ± 0.39
VS (%)	85.55 ± 6.49	2.65 ± 0.18
C/N (soild)	12.31 ± 1.26	8.51 ± 0.89
pH	7.21	8.10
Total alkalinity (mg CaCO_3_/L)	1825 ± 132	15,732 ± 759
NH_3_-N (mg·L^−1^)	1704 ± 126	3638 ± 263
SCFAs (mg·L^−1^)	5683 ± 397	4812 ± 365

Note: VS: content of volatile solids; TS: content of total solids.

**Table 2:** Chemical and physical and characteristics of hydrochar.

Properties	Hydrochar
pH	5.72 ± 0.1
Average pore size (nm)	3.61 ± 0.26
Total pore volume (cm^3^·g^−1^)	0.41 ± 0.03
Specific surface area (cm^3^·g^−1^)	123.88 ± 7.65
VM (dry basis) (wt %)	58.25 ± 4.38
FC (dry basis) (wt %)	36.94 ± 1.47
Ash (dry basis) (wt %)	3.355 ± 0.21
C (dry basis) (wt %)	47.61 ± 3.99
H (dry basis) (wt %)	5.26 ± 0.31
N (dry basis) (wt %)	3.76 ± 0.27
Conductivity (mS·cm^−1^)	0.47 ± 0.04

Note VM: content of volatile matter, FC: content of fixed carbon, Ash: content of ash.

## Data Availability

The original contributions presented in this study are included in the article. Further inquiries can be directed to the corresponding authors.

## Supplementary Materials

The following supporting information can be downloaded at: https://www.mdpi.com/article/10.3390/ma19081563/s1. Table S1: Relative abundance of bacterial and archaeal communities, (a) Relative abundance of the top 10 bacteria at phylum level, (b) Relative abundance of the top 15 bacteria at genus level, (c) Relative abundance of archaea at phylum level, (d) Relative abundance of the top 10 archaea at genus level; Table S2 Hydrolysis, acidogenesis and acetogenesis pathways and associated genes; Table S3 Methanogenesis pathways and associated genes.

## References

[1] Wan S., Xi B., Xia X., Li M., Lv D., Wang L., Song C. (2012). Using fluorescence excitation–emission matrix spectroscopy to monitor the conversion of organic matter during anaerobic co-digestion of cattle dung and duck manure. Bioresour. Technol..

[2] Hou S., Liu L. (2025). Waterfowl industry and technology development report of 2024. Chin. J. Anim. Sci..

[3] Shi J.-C., Liao X.-D., Wu Y.-B. (2010). Methane generation during anaerobic fermentation of four livestock slurries: Methane generation during anaerobic fermentation of four livestock slurries. Chin. J. Eco-Agric..

[4] Wang C., Wang Y., Wang H., Xu L. (2023). Analysis on seasonal variation of meat duck manure excretion and characteristics in large-scale farms. China Poult..

[5] Yan T., Zhu Z., Gao L., Lu L., Sun H., Yu X., Luo Q. (2020). Seasonal variation in emission characteristics of meat duck manure. Chin. J. Eco-Agric..

[6] Liu T., Sung S. (2002). Ammonia inhibition on thermophilic aceticlastic methanogens. Water Sci. Technol..

[7] Jiang Y., McAdam E., Zhang Y., Heaven S., Banks C., Longhurst P. (2019). Ammonia inhibition and toxicity in anaerobic digestion: A critical review. J. Water Process Eng..

[8] He A., Liu Y., Cao C., Xiao Q., Xu J., Feng Y., Zhang Z., Xi Y. (2025). Humic acid–anchored hydrochar for enhancing methane production in anaerobic digestion of cow manure. J. Environ. Manag..

[9] Yenigün O., Demirel B. (2013). Ammonia inhibition in anaerobic digestion: A review. Process Biochem..

[10] Ye M., Liu J., Ma C., Li Y.-Y., Zou L., Qian G., Xu Z.P. (2018). Improving the stability and efficiency of anaerobic digestion of food waste using additives: A critical review. J. Clean. Prod..

[11] Sugiarto Y., Sunyoto N.M.S., Zhu M., Jones I., Zhang D. (2021). Effect of biochar addition on microbial community and methane production during anaerobic digestion of food wastes: The role of minerals in biochar. Bioresour. Technol..

[12] Wang X., Zhao J., Yang Q., Sun J., Peng C., Chen F., Xu Q., Wang S., Wang D., Li X. (2017). Evaluating the potential impact of hydrochar on the production of short-chain fatty acid from sludge anaerobic digestion. Bioresour. Technol..

[13] Ali M., Elaziz M.A., Fujii M., Elsheikh A.H. (2025). Advanced machine learning-based prediction of biogas production boosted by functionalized nanomaterials using the snow geese algorithm. J. Environ. Chem. Eng..

[14] Shi Z., Liu S., Wang S., Niedzwiecki L., Baranowski M., Czerep M., Tang C., Kawi S., Wang C.-H., Jiang J. (2023). Hydrothermal carbonization coupled with pyrolysis: An innovative approach to digestate management. Green Energy Resour..

[15] Usman M., Shi Z., Ren S., Ngo H.H., Luo G., Zhang S. (2020). Hydrochar promoted anaerobic digestion of hydrothermal liquefaction wastewater: Focusing on the organic degradation and microbial community. Chem. Eng. J..

[16] Odales-Bernal L., González L.M.L., Ghysels S., Lobanov V., De Vrieze J., Barrera E.L., Ronsse F. (2025). Optimized hydrothermal carbonization of chicken manure and anaerobic digestion of its process water for better energy management. J. Environ. Manag..

[17] Usman M., Shi Z., Ji M., Ren S., Luo G., Zhang S. (2021). Microbial insights towards understanding the role of hydrochar in alleviating ammonia inhibition during anaerobic digestion. Chem. Eng. J..

[18] Choe U., Mustafa A.M., Lin H., Xu J., Sheng K. (2019). Effect of bamboo hydrochar on anaerobic digestion of fish processing waste for biogas production. Bioresour. Technol..

[19] Kundu R., Kunnoth B., Pilli S., Polisetty V.R., Tyagi R.D. (2023). Biochar symbiosis in anaerobic digestion to enhance biogas production: A comprehensive review. J. Environ. Manag..

[20] Wang T., Zhang D., Dai L., Dong B., Dai X. (2018). Magnetite triggering enhanced direct interspecies electron transfer: A scavenger for the blockage of electron transfer in anaerobic digestion of high-solids sewage sludge. Environ. Sci. Technol..

[21] Chen Y., Wang Y., Xie H., Cao W., Zhang Y. (2023). Varied promotion effects and mechanisms of biochar on anaerobic digestion (AD) under distinct food-to-microorganism (F/M) ratios and biochar dosages. Waste Manag..

[22] Martins G., Salvador A.F., Pereira L., Alves M.M. (2018). Methane production and conductive materials: A critical review. Environ. Sci. Technol..

[23] Chen Y., Song W., Hong T., Zhang L., Ding D., Ruan H. (2026). Research progress in reparation of functional materials for ater treatment using agricultural and forestry wastes. Environ. Eng..

[24] Hurst G., Ruiz-Lopez S., Rivett D., Tedesco S. (2022). Effect of hydrochar from acid hydrolysis on anaerobic digestion of chicken manure. J. Environ. Chem. Eng..

[25] Xu J., Mustafa A.M., Lin H., Choe U.Y., Sheng K. (2018). Effect of hydrochar on anaerobic digestion of dead pig carcass after hydrothermal pretreatment. Waste Manag..

[26] Ren S., Usman M., Tsang D.C.W., O-Thong S., Angelidaki I., Zhu X., Zhang S., Luo G. (2020). Hydrochar-facilitated anaerobic digestion: Evidence for direct interspecies electron transfer mediated through surface oxygen-containing functional groups. Environ. Sci. Technol..

[27] Yu M., Shao H., Wang P., Ren L. (2024). Metagenomic analysis reveals the mechanisms of biochar supported nano zero-valent iron in two-phase anaerobic digestion of food waste: Microbial community, CAZmey, functional genes and antibiotic resistance genes. J. Environ. Manag..

[28] He J., Luo T., Shi Z., Angelidaki I., Zhang S., Luo G. (2022). Microbial shifts in anaerobic digestion towards phenol inhibition with and without hydrochar as revealed by metagenomic binning. J. Hazard. Mater..

[29] Yang Y., Wang M., Yan S., Yong X., Zhang X., Awasthi M.K., Xi Y., Zhou J. (2023). Effects of hydrochar and biogas slurry reflux on methane production by mixed anaerobic digestion of cow manure and corn straw. Chemosphere.

[30] Masís-Meléndez F., Segura-Chavarría D., García-González C.A., Quesada-Kimsey J., Villagra-Mendoza K. (2020). Variability of physical and chemical properties of TLUD stove derived biochars. Appl. Sci..

[31] He J., Ren S., Zhang S., Luo G. (2021). Modification of hydrochar increased the capacity to promote anaerobic digestion. Bioresour. Technol..

[32] Shi Z., Usman M., He J., Chen H., Zhang S., Luo G. (2021). Combined microbial transcript and metabolic analysis reveals the different roles of hydrochar and biochar in promoting anaerobic digestion of waste activated sludge. Water Res..

[33] American Public Health Association (1995). Standard Methods for the Examination of Water and Wastewater.

[34] Martin M. (2011). Cutadapt removes adapter sequences from high-throughput sequencing reads. EMBnet J..

[35] Wood D.E., Lu J., Langmead B. (2019). Improved metagenomic analysis with kraken 2. Genome Biol..

[36] Li D., Liu C.-M., Luo R., Sadakane K., Lam T.-W. (2015). MEGAHIT: An ultra-fast single-node solution for large and complex metagenomics assembly via succinct *de bruijn* graph. Bioinformatics.

[37] Steinegger M., Söding J. (2017). MMseqs2 enables sensitive protein sequence searching for the analysis of massive data sets. Nat. Biotechnol..

[38] Hyatt D., Chen G.-L., LoCascio P.F., Land M.L., Larimer F.W., Hauser L.J. (2010). Prodigal: Prokaryotic gene recognition and translation initiation site identification. BMC Bioinf..

[39] Liao Y., Smyth G.K., Shi W. (2014). FeatureCounts: An efficient general purpose program for assigning sequence reads to genomic features. Bioinformatics.

[40] Shen Y., Linville J.L., Urgun-Demirtas M., Schoene R.P., Snyder S.W. (2015). Producing pipeline-quality biomethane via anaerobic digestion of sludge amended with corn stover biochar with in-situ co2 removal. Appl. Energy.

[41] Shi Z., Campanaro S., Usman M., Treu L., Basile A., Angelidaki I., Zhang S., Luo G. (2021). Genome-centric metatranscriptomics analysis reveals the role of hydrochar in anaerobic digestion of waste activated sludge. Environ. Sci. Technol..

[42] Yuan Y., Hu X., Chen H., Zhou Y., Zhou Y., Wang D. (2019). Advances in enhanced volatile fatty acid production from anaerobic fermentation of waste activated sludge. Sci. Total Environ..

[43] Li Y., Xu H., Hua D., Zhao B., Mu H., Jin F., Meng G., Fang X. (2020). Two-phase anaerobic digestion of lignocellulosic hydrolysate: Focusing on the acidification with different inoculum to substrate ratios and inoculum sources. Sci. Total Environ..

[44] Shen Y., Zhang X., Ye M., Zha X., He R. (2024). Effects of fe-modified digestate hydrochar at different hydrothermal temperatures on anaerobic digestion of swine manure. Bioresour. Technol..

[45] Mostafa A., Im S., Lee M.-K., Song Y.-C., Kim D.-H. (2020). Enhanced anaerobic digestion of phenol via electrical energy input. Chem. Eng. J..

[46] Brown J.W. (2016). Principles of Microbial Diversity.

[47] Yadav M., Joshi C., Paritosh K., Thakur J., Pareek N., Masakapalli S.K., Vivekanand V. (2022). Organic waste conversion through anaerobic digestion: A critical insight into the metabolic pathways and microbial interactions. Metab. Eng..

[48] Wang C., Qiao W., Chen H., Xu X., Zhu L. (2019). A short-term stimulation of ethanol enhances the effect of magnetite on anaerobic digestion. Appl. Microbiol. Biotechnol..

[49] Baek G., Kim J., Lee C. (2016). A long-term study on the effect of magnetite supplementation in continuous anaerobic digestion of dairy effluent—Enhancement in process performance and stability. Bioresour. Technol..

[50] Ou Y.-F., Dong H.-P., McIlroy S.J., Crowe S.A., Hallam S.J., Han P., Kallmeyer J., Simister R.L., Vuillemin A., Leu A.O. (2022). Expanding the phylogenetic distribution of cytochrome *b*-containing methanogenic archaea sheds light on the evolution of methanogenesis. ISME J..

[51] Wang H., Byrne J.M., Liu P., Liu J., Dong X., Lu Y. (2020). Redox cycling of Fe(II) and Fe(III) in magnetite accelerates aceticlastic methanogenesis by *Methanosarcina mazei*. Environ. Microbiol. Rep..

[52] Wang Y., Zhang J., Li Y., Jia S., Song Y., Sun Y., Zheng Z., Yu J., Cui Z., Han Y. (2020). Methane production from the co-digestion of pig manure and corn stover with the addition of cucumber residue: Role of the total solids content and feedstock-to-inoculum ratio. Bioresour. Technol..

[53] Ren S., Tong Z., Yong X., Xi Y., Liu F., Zhou J. (2024). The new strategies of using nitrogen and iron modified hydrochar to enhance methane production during co-anaerobic digestion of cow manure and corn straw. J. Environ. Chem. Eng..

[54] Cheng Q., Xu C., Huang W., Jiang M., Yan J., Fan G., Zhang J., Chen K., Xiao B., Song G. (2020). Improving anaerobic digestion of piggery wastewater by alleviating stress of ammonia using biochar derived from rice straw. Environ. Technol. Innov..

[55] Li J., Zhou L., Zhao J., Zhang W., Pan B., Hua M. (2025). Enhanced methanogenesis of wastewater anaerobic digestion by nanoscale zero-valent iron: Mechanism on intracellular energy conservation and amino acid metabolism. Bioresour. Technol..

